# Fisheries ecological knowledge, FEK: Contribution to the knowledge of the ecology and distribution of houndsharks and dogfish shark (family Triakidae and Squalidae) in the Canary Islands

**DOI:** 10.1371/journal.pone.0344910

**Published:** 2026-03-18

**Authors:** Rosario Luque Cabrera, Ana Espino-Ruano, José Juan Castro, Airam Guerra-Marrero, Lorena Couce-Montero, Raibel Núñez-González, Tomàs Bañeras, David Jiménez-Alvarado

**Affiliations:** 1 IU-ECOAQUA, University of Las Palmas de Gran Canaria, Las Palmas de Gran Canaria, Spain; 2 University of Atlántico Medio, Las Palmas, Spain; Universidade Federal do Rio de Janeiro, BRAZIL

## Abstract

Currently, it is assumed that most of elasmobranchs (sharks, rays and skates) had suffered a high depletion in their populations worldwide, but there is an important lack of biological and fishing information over the bulk of the species that do not permit to determine the real status of their populations. Seven of these scarce information species are the houndsharks and dogfish shark (family Triakidae and Squalidae) found in the Canary Islands, which actual status level and contribution to the catch in the archipelago are still unknown. 136 interviews were carried out among artisanal fishers from all the islands, in which very few of them were able to distinguish all the species. Two houndshark and one dogfish shark species were frequently identified and/or caught (the common smoothhound (*Mustelus mustelus*), the tope shark (*Galeorhinus galeus*)*,* and the shortnose spurdog (*Squalus megalops*)), and half of the fishers were not able to distinguish the sex of these fishes. The houndsharks and dogfish shark are preferentially distributed down to 200 metres depth, with higher catches off the eastern and central islands. These sharks are caught all year round with highest peaks during the summer months, probably coinciding with aggregations for mating and reproduction in shallower waters. Houndsharks and dogfish shark are generally not targeted by artisanal fisheries and, although sometimes landed, most of the catch is usually discarded. Sharks caught range from 100 to 120 cm in total length for common smoothhound and tope sharks, while the catch size for shortnose spurdog varies between 50 and 60 cm in total length. Improving taxonomic knowledge within the fishing sector is essential for sustainable management and conservation of houndshark and dogfish shark species in Canarian waters.

## 1. Introduction

Elasmobranchs (sharks, rays, and skates) populations are currently in a highly depleted state, with a quarter of them threatened and a third of them listed as Critically Endangered due to anthropogenic causes, such as overfishing and habitat destruction [1,2]. In fact, since the 1970s, some of these species have shown a high reduction of their populations up to 71% [3].

A high biodiversity of elasmobranchs is found in the Canary Islands [4,5] with approximately 86 species reported [6]. For the shark species of the family Triakidae present in this region, the most abundant is *Mustelus mustelus* [7–9] popularly known as “cazón”. In the Canary Islands, these sharks are mainly caught using a gillnet known as cazonal, but they are also fished with trammel nets, bottom longlines, and/or handline.

The actual contribution of sharks and rays to total catches in the Canary Islands is still unknown and are under-reported or misreported in official records [10–12]. As a result, there are inaccuracies in fisheries statistics concerning species identification, which is likely to be even more noticeable for those sharks whose taxonomic identification is difficult for non-experts. This overall problem results in the lumping of several species into the same category [13,14], and frequently, available data on landings and fishing effort are incomplete and/or inadequate [14].

This paper analyses seven species of sharks belonging to two families. On the one hand, the family Triakidae, of the order Carcharhiniformes, includes four species fished in the Canary Islands, such as *Galeorhinus galeus* (Linnaeus, 1758), *Mustelus asterias* (Cloquet, 1819), *Mustelus mustelus* (Linnaeus, 1758), and *Mustelus punctulatus* (Risso, 1827). On the other hand, in the family Squalidae, order Squaliformes, there are three species that are fished more or less frequently in this region, *Squalus acanthias* (Linnaeus, 1758), *Squalus blainville* (Risso, 1827), and *Squalus megalops* (Macleay, 1881).

Common smoothhound (*Mustelus mustelus*) accounts for 52.94% of the catches of houndshark off the islands [9,15], while catches of dogfish shark by recreational fishers are rare [9,16]. The blackspotted smoothhound (*M. punctulatus*) is often confused with common smoothhound (*M. mustelus*) and may even be mistakenly recorded as such [17]. The same problem appears for longnose spurdog (*Squalus blainville*) and shortnose spurdog (*S. megalops*), not only because of their similar size and coloration, but also because they tend to frequent the same bathymetric range [17,18].

In this context, the aims of this study were: (1) To collect biological and fisheries information on the seven shark species through citizen science techniques, (2) to assess the degree of knowledge of artisanal fishers regarding the identification of these shark species, and (3) to make a first approximation of the status of houndsharks and dogfish shark stocks in island waters.

## 2. Materials and methods

### 2.1. Study area

**Fig 1:**
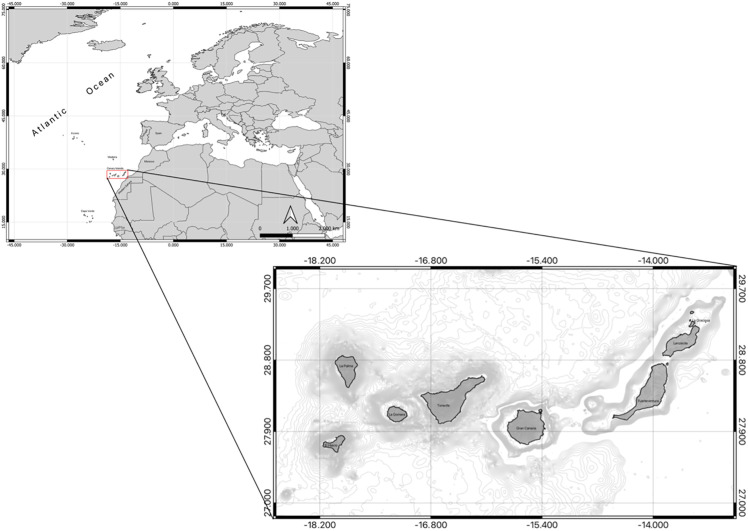
Location of the Canary Islands.

The Canary Islands Archipelago is located in the Central Eastern Atlantic Ocean, approximately 100 km from the west African coast (Fig 1). With a surface area of 7,501 km^2^, it is made up of eight oceanic islands (Lanzarote, Fuerteventura, Gran Canaria, Tenerife, El Hierro, La Gomera, La Palma and La Graciosa) (Fig 1). The islands are separated between each other and with the African continental shelf by depths between 1200 and 3000 m, except for Fuerteventura, Lanzarote and La Graciosa that share the same insular shelf. The oldest islands are the eastern ones (La Graciosa, Lanzarote, Fuerteventura) and the more recent are the western ones (Gomera, La Palma and El Hierro), where the two last have had volcanic eruptions in the last 15 years.

### 2.2. Data collection

To collect the information, artisanal fishers were interviewed in their respective fishers’ associations in each of the islands of the archipelago. The surveys were carried out between February and June 2023, making a total of 38 questions that were structured in four thematic blocks:

1. Description of the fishery (fishing systems, fishing grounds, and seasonality of the fishing activity).
2. Spatial distribution, biology, and behaviour of the species.
3. Catch levels and their evolution in the medium term.
4. Conservation status of species and habitats.

### 2.3. Data analyses

**Fig 2:**
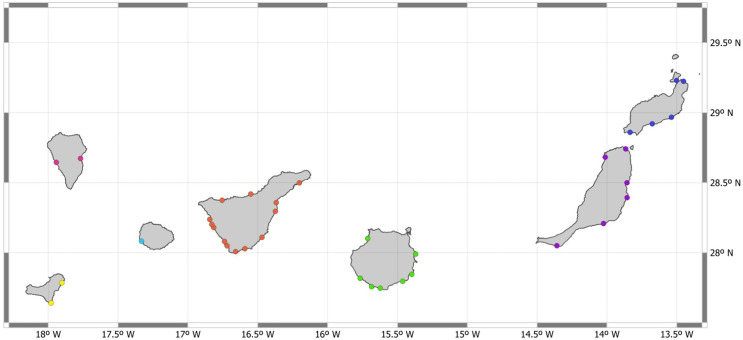
Fishers’ guilds surveyed throughout the archipelago.

A total of 136 artisanal fishers were interviewed across the archipelago (Fig 2). All data was pre-analyses using Shapiro-Wilk and Levene test to assess normality and homoscedasticity. Different analyses of variance (ANOVA) and non-parametric (Kruskall-Wallis) tests were carried out to establish whether there were significant differences in shark sex, occurrence, and time of day when catches were made. Moreover, interviews were also addressed to know if these sharks are targeting species or part of the by-catch, and when, and which fishing gears are used. Additionally, a t-test was carried out to check if there were significant differences concerning the species that were caught together with houndsharks and dogfish shark. All the tests were performed on R Studio (R Core Team, 2023).

For the three species that were caught the most, their distribution in the Canary Islands was determined using Jenks’ method of natural breaks. Finally, mosaic graphs and the corresponding Chi-square test of independence were carried out to determine the total lengths of the sharks that were frequently caught, as well as to observe whether there were significant differences throughout the archipelago.

Georeferenced data on interactions with houndsharks and dogfih shark were processed using QGIS version 3.40. Spatial interpolation and frequency mapping were performed using the “Heatmap (Kernel Density Estimation)” plugin, which generates a raster layer based on point density calculated via kernel estimation. (Fig 4) A Gaussian distribution was assumed, with density values decreasing gradually with distance from each point, and a 15 km search radius was applied, highlighting areas with higher concentrations of interaction records through higher pixel intensity, enabling the visualization of spatial distribution patterns.

## 3. Results

**Fig 3:**
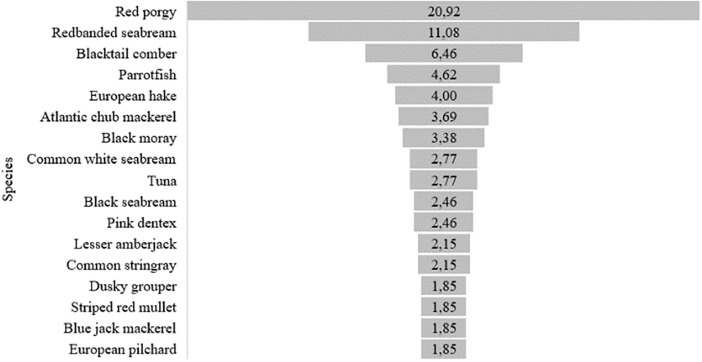
Percentages of the most common fish species caught in the artisanal fishery where houndsharks and dogfish shark is a co-occurring species.

According to the information collected from the surveys, 75% of the fishers could not clearly distinguish the seven species of houndsharks and dogfish shark present in the Canary Islands. The common smoothhound (*Mustelus mustelus*), the tope shark (*Galeorhinus galeus*), and the shortnose spurdog (*Squalus megalops*) are the most easily identifiable species by fishers. Likewise, around 98% of the fishers consider the catches of these sharks as incidental, and only 2.2% stated that they are occasionally targeted species as part of a multispecies fishery. In fact, of the 14 non-elasmobranch most common species that are caught together with houndsharks and dogfish shark, making up 70.8% of the total catch landed by the fishers surveyed, the red porgy (*Pagrus pagrus*) (t-test, *p*-value = 9.1-10^-5^) (Fig 3).

The houndsharks and dogfish shark are caught all year round, although they are more frequent in the summer months, possibly coinciding with the spawning or reproductive season of the species.

**Fig 4:**
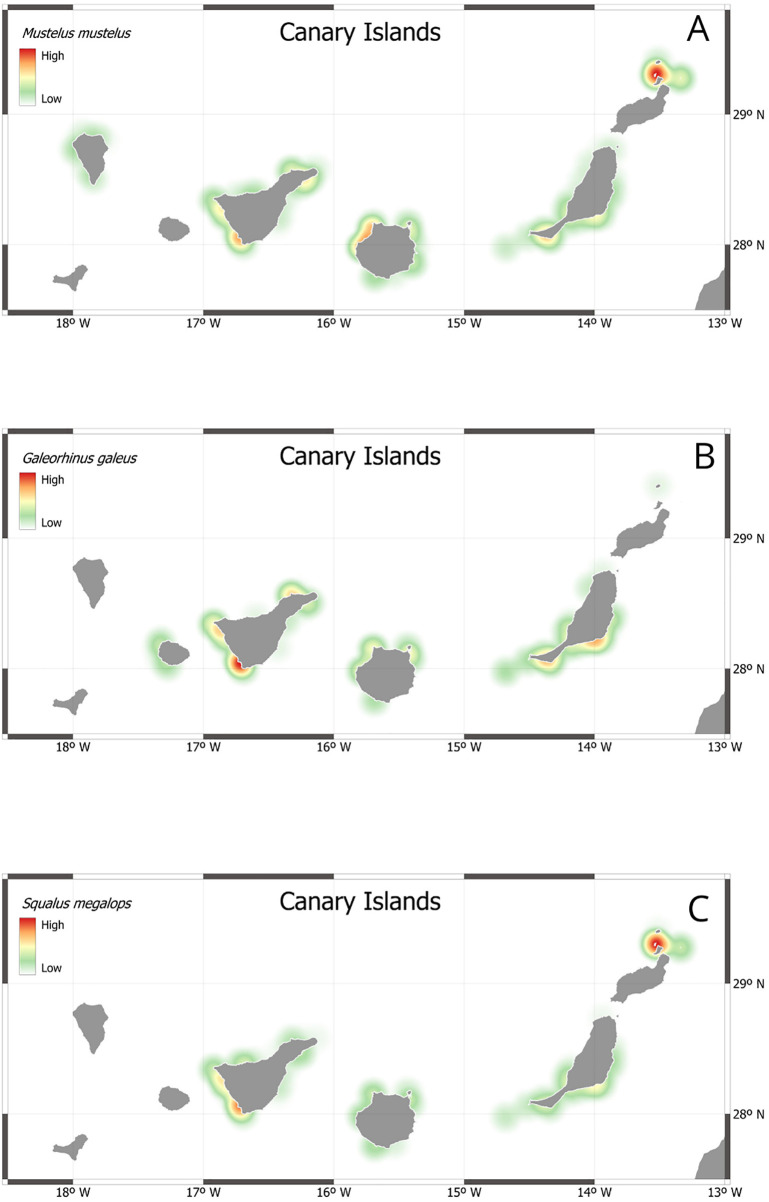
Species spatial distribution heatmap in the archipelago related to fishers’ quiestionnaires: A) *Mustelus mustelus*, B) *Galeorhinus galeus*; C) *Squalus megalops*.

After studying the data provided by artisanal fishers, differences in the geographical distribution of encounters throughout the Canary Islands can be observed. Houndsharks and dogfish shark have a greater presence in the eastern (La Graciosa, Lanzarote and Fuerteventura) and central islands (Gran Canaria and Tenerife), being less abundant in the western islands (La Palma, La Gomera and El Hierro), which may be because these last islands are geologically more recent and rocky bottoms are much more abundant than sandy ones, which are the preferred by these species (Fig 4). In addition, the greater knowledge on these species shown by fishers from the eastern and central islands of the archipelago put also into evidence that houndsharks and dogfish shark are more frequent and/or abundant on these islands, with a greater diversity of species in the catches. Fishers from the westernmost islands (La Palma and La Gomera) only reported the presence of the common smoothhound (*Mustelus mustelus)* in their catches and, from the responses obtained, it appears that none of these species are present on the island of El Hierro, at least not as part of the commercial catches.

**Fig 5:**
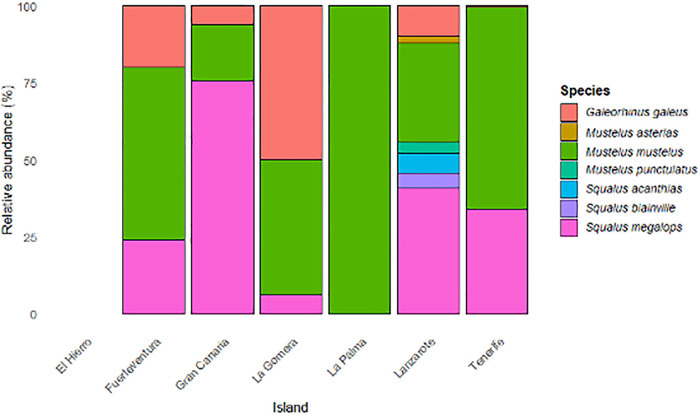
Relative abundance of houndsharks and dogfish in the Canary Islands.

The relative abundance analysis shows notable variations in the specific composition of houndsharks and dogfishs between the different islands of the Canary archipelago (Fig 5). *Mustelus mustelus* was the dominant species on most islands, reaching proportions of over 70% in La Palma, Tenerife, and Gran Canaria. *Squalus megalops* and *Galeorhinus galeus* are particularly common in Fuerteventura, Gran Canaria, Tenerife, La Gomera, and Lanzarote. In contrast, *Mustelus asterias*, *M. punctulatus*, *Squalus acanthias* and *S. blainville* showed much lower abundances and restricted distributions, mainly in Lanzarote, the island with the greatest species diversity.

The surveyed species are mainly caught and/or observed in a depth range from the shoreline to 200 metres, except for *Mustelus asterias*, *Squalus acanthias* and *S. blainville,* which are caught at greater depths (preferably from 200 metres downwards). None of the respondents reported catches of the three *Mustelus* spp. and *S. acanthias* at depths greater than 500 metres.

**Fig 6:**
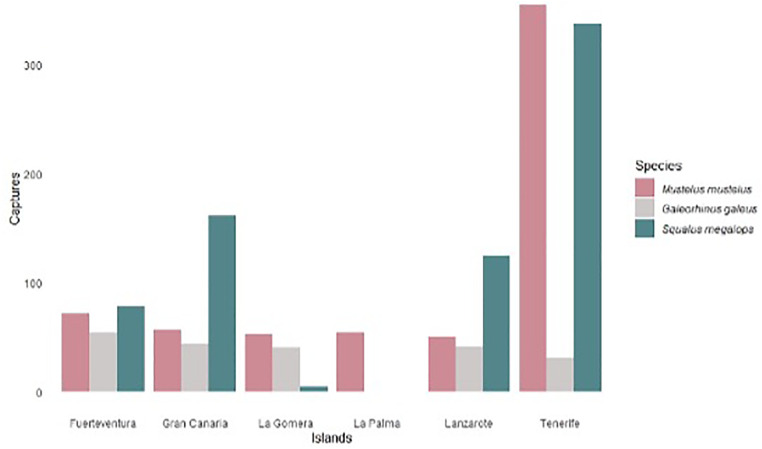
Catches (kg) of the three most abundant species among Canarian fishers for each Canary Islands. Data referring to 2022.

Although no significant differences were found (Kruskall-Wallis, *p*-value = 0.406) in the hours of capture (24.4% in the morning, 27.1% at night, and 25.78% at dawn), there was variability in the occurrence of catches. On the one hand, common smoothhound (*Mustelus mustelus*) and shortnose spurdog (*Squalus megalops*) are the species most caught in the islands. In the case of common smoothhound they refer to catches that are mostly subsequently sold, and in the case of shortnose spurdog they refer to accidental catches, especially when longline fishing is used. On the other hand, the common smoothhound and the tope shark (*Galeorhinus galeus*) were the most abundant in the artisanal landing with a total of 368.78 ± 1780.20 kg and 31.45 ± 42.25 kg in 2022, respectively. The standard deviation differs from the mean due to the dispersion of catch data among fishermen (Fig.6). The island of El Hierro is excluded since the fishermen surveyed do not catch these species and have not even seen them.

**Fig 7:**
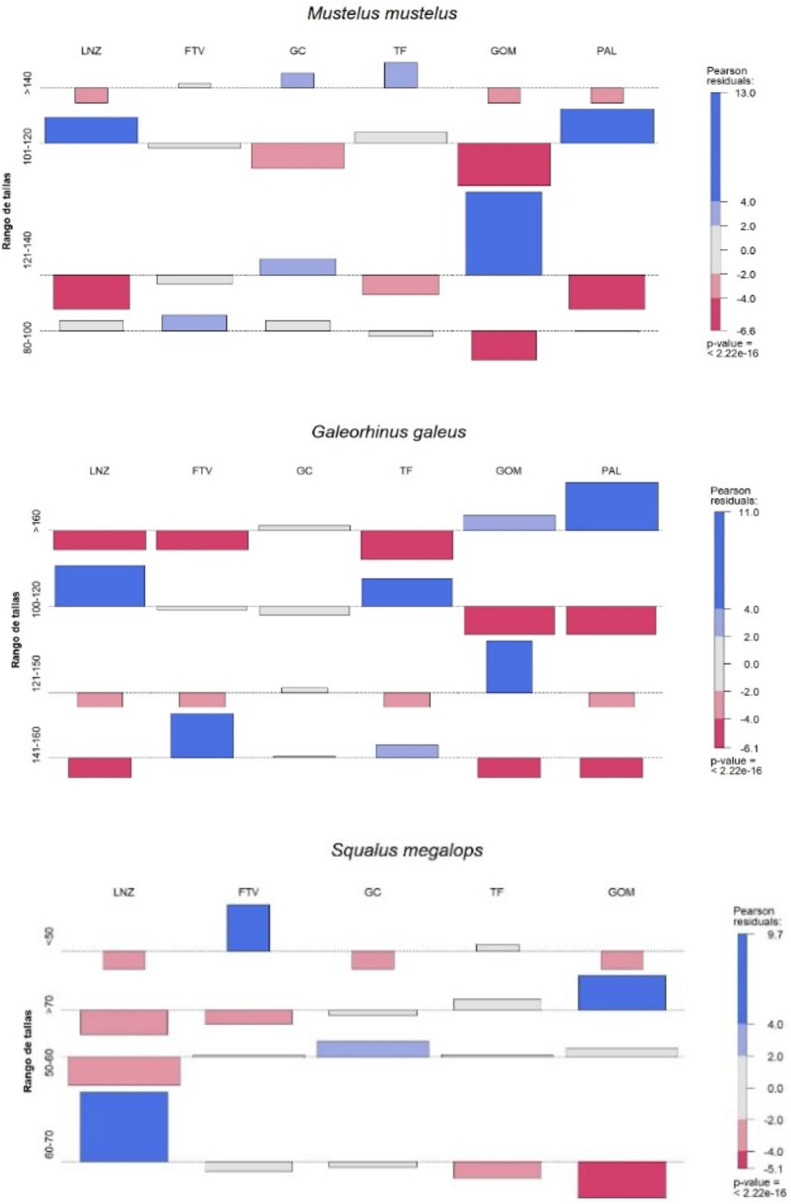
Total length range for each island and Chi-square result for the species archipelago: A) *Mustelus mustelus*, B) *Galeorhinus galeus*, C) *Squalus megalops* (LNZ – Lanzarote, FTV – Fuerteventura, GC – Gran Canaria, TF – Tenerife, GOM – La Gomera, PAL – La Palma).

Of the three most common species, a wide size range is captured. Thus, for *Mustelus mustelus* and *Galeorhinus galeus,* the most frequent sizes (total length, TL) reported are in the range 100–120 cm (45.24% and 44.29%, respectively), while for *Squalus megalops,* fishers gave a range between 50–60 cm TL (43.93%). All of them show significant differences not only within the same island, but also within the archipelago (Fig 7).

**Fig 8:**
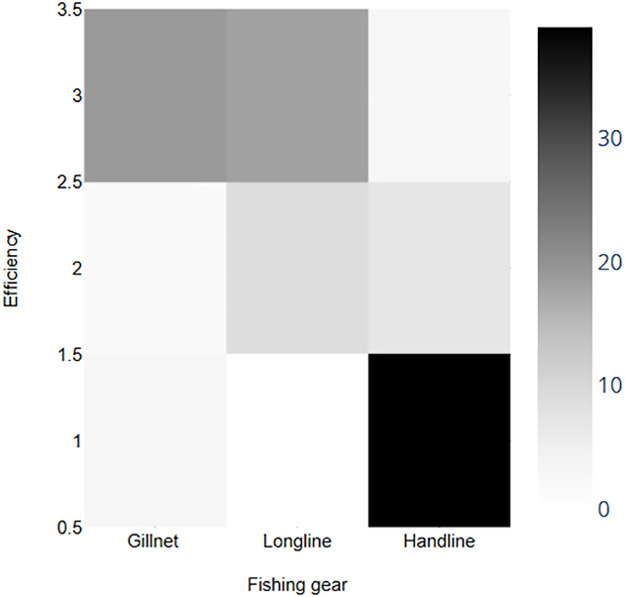
Frequency accumulation of answers about the efficiency of each fishing gear being 1: Low efficiency, 2: Medium efficiency and 3: High efficiency.

Table 1 summarizes the permitted methods on each island to fish houndsharks, and in based on the degree of efficiency given by fishers of the different fishing system (low/medium/high) used, there are significant differences between handline, longline and the gillnet (Kruskal-Wallis ANOVA; *p*-value = 1.281^-13^). The gillnet is the most efficient fishing gear, followed by the longline on those islands where the former is prohibited; and finally, the handline is the least efficient system for catching these species (Fig 8). In addition, 68.6% of the respondents reported aggregatory behaviour in the species, particularly in shortnose spurdog (*Squalus megalops*), and 91.0% of them stated that the aggregation of specimens increases the efficiency of the gear they use.

**Table 1:** Permits (✔) and prohibitions (×) for the fishing gear used in this study on each island of the archipelago.

Island	Handline	Longlines	Gillnet (cazonal)
Lanzarote	✔	✔	×
Fuerteventura	✔	×	×
Gran Canaria	✔	✔	✔
La Graciosa	✔	×	×
Tenerife	✔	✔	✔
La Gomera	✔	×	×
La Palma	✔	✔	✔
El Hierro	✔	**×**	**×**

Even though some fishers have claimed that during the full moon phase, there is a greater presence of females, 31.2% of fishers claim that they usually catch specimens of both sexes. It is important to note that 48.8% of fishers admit that they do not know how to distinguish between males and females, while others say that they catch more females (17.6%).

As far as landings are concerned, only 27.3% of the fishers land common smoothhound and 20% of the fishers land tope shark; meanwhile, most of them discard these species (38.2% and 40%, respectively). Shortnose spurdog is the most discarded species, accounting for 61.2% of the catches. This seems to be related to the lack of market for these species and/or their low selling price.

If we consider the fishers’ perception of the evolution of the catches of these species over the last 5 years, 50.9% of them have observed an increase in their captures, compared to the 17.86% who have observed a decrease, and the 31.3% who think that do not change have occurred in this lapse. However, although fishing effort (fishing days) has remained almost constant in recent 5 years, fishers consider that tope shark and shortnose spurdog are being caught more per unit of effort (35.6% and 47.4%, respectively); while 42.2% report a decrease in common smoothhound abundance. Even so, respondents consider that the stock of each of these species in Canarian waters is not overexploited, considering a priori one stock per species for the whole archipelago. An in-depth acoustic and tagging study will be necessary to verify the latter.

Accordingly, although fishers may have noticed a greater abundance of shortnose spurdog (*S. megalops*) on days when the moon is full or when specimens come closer to the coast in summer, they have not evidenced a significant displacement of the species to other areas. Therefore, 86.7% of the fishers consider that the habitats of these sharks are not altered, which is why, in the case of migration or displacement at certain times of the life cycle, it could be related to the breeding season. However, some fishers who consider that the populations of these sharks have shifted, with anthropogenic causes being one of them (marine pollution, etc.), together with water temperature (climate change), referring to the fact that they are nowadays found in shallower waters.

## 4. Discussion

One of the non-invasive ways of obtaining information on the state of fishery resources is through fishers and their ecological knowledge, that provide valuable insights for fisheries research and management, which is very valuable in cases where scientific data are scarce [16,19].

In line with what has been described in several studies, there are problems in the identification of species due to the morphological similarities they present, especially among species of the genus *Mustelus* [20–24]. As a result, 75% of fishers do not differentiate between the seven species of houndsharks and dogfish sharks present in the Canarian waters, only three of which are frequently identified as common smoothhound (*Mustelus mustelus*), tope shark (*Galeorhinus galeus*) and shortnose spurdog (*Squalus megalops*). At the same time, as they are not considered target species due to their low sale price, the available biological and fishing information is very scarce. Curiously, in four of the seven islands the use of the gillnet, the most efficient fishing gear for catching these sharks, is not allowed, not due to the overfished status of houndshark and dogfish sharks’ populations, but for its impact on other more valuable fish species like the black seabream (*Spondyliosoma cantharus*). In addition, many fishers do not use it because its use is restricted or prohibited in their usual fishing grounds.

The information obtained in the surveys shows a very heterogeneous spatial distribution pattern of these species throughout the archipelago, with a greater presence in the eastern and central islands, which coincides with what Espino *et al*., (2022) reported. Similar is the case for other benthic shark and ray species such as *Squatina squatina* and *Gymnura altavela* [5,25]. The apparent geographic distribution of the species could be related to the steep slope and ruggedness of the island shelves of the westernmost islands, with sparse sandy areas where these species tend to feed. These sharks show a clear preference for sandy and mixed (sandy-rocky) bottoms, habitats much more frequent in the easternmost part of the archipelago [25,26].

The results obtained show an east-west ecological gradient in the structure of dogfish communities in the Canary Islands. The eastern islands showed greater species richness and a more equitable distribution of abundance, while the western islands had impoverished communities or even no individuals at all. The overall dominance of *Mustelus mustelus* suggests high ecological plasticity, allowing it to occupy a wide variety of benthic habitats, from shallow sandy bottoms to deeper areas [20,27]. The coexistence of multiple species in Lanzarote suggests that habitat heterogeneity and niche availability play a key role in the structuring of local communities [28].

The three most known species by fishers (*Mustelus mustelus*, *Galeorhinus galeus,* and *Squalus megalops*) are mostly caught in the first 200 metres of the water column, according to the vertical distribution described in the literature [18,29,30]. Probably, these species have a deeper distribution, as described by Triay *et al.* (2023) who reported *G. galeus* at depths of 800 m, but the commercial fishing does not go much deeper than 200 m because their abundance decreases significantly below this threshold.

Although these three species are caught at all sizes, they are indeed more frequent when the total length is between 100–120 cm for *Mustelus mustelus* and *Galeorhinus Galeus,* and between 50–60 cm for *Squalus megalops*. It should be noted that they have no minimum catch size in the Canary Islands, so if we emphasize the proposal of the same made in the study by González *et al*. (2012) [31] (96 cm, 140 cm and 66 cm, respectively), *M. mustelus* is the only species that normally is caught as an adult, while the other two are likely to be juveniles and/or sub-adults.

On the other hand, the aggregation of juvenile and sub-adult houndsharks and dogfish sharks seems to occur in late spring (May-June) and early summer (July-August) [9]. Considering that the highest peak of catches occurs in summer, it could explain the catches of *Galeorhinus galeus* and *Squalus megalops* at early sizes. In this sense, Elisio *et al*. (2016) [32] indicate that *Mustelus mustelus* perform reproductive migrations with aggregations of individuals in shallower areas associated with warm water masses. Likewise, adults tend to aggregate in shallow waters at the end of the summer for possible mating [9], which could explain why 31.2% of fishers stated that they capture both females and males. In any case, it should be noted that even more fishers do not know how to differentiate the sex of individuals and that these results could be biased by the very nature of the surveys as they were carried out on a specific group.

Most observations of elasmobranchs take place at night when they are in shallower waters, either for feeding or as a refuge from predators, reflecting a possible circadian migration since different studies have shown that during the day most individuals are in deeper waters [33–36]. In contrast to our study in which surveys reveal that both family species are caught mainly at dawn and dusk, coinciding with the fishers’ schedule, and that the concentration of adult sharks in shallow waters occurs in the summer months, as also indicated by the studies of Da Silva *et al*., (2013) [37] and Espino *et al*., (2022) [9]. This statement supports the information given by a large proportion of fishers (68.64%) on the presence of aggregations of shortnose spurdog (*Squalus megalops*), especially females, in relatively shallow waters on nights during full moon, which could be related to the laying or release of young. Similarly, this behaviour has been described for *Mustelus mustelus* in South African waters with concentrations of individuals during the summer in shallow waters of both a coastal lagoon and harbour areas, well-marked by water temperature (between 18 and 22 ºC), and not by the lunar phase [38].

At population level, the results showed an increase in stocking for the best-known species (common smoothhound, tope shark and shortnose spurdog) in recent years, as also indicated by some reports for other areas in relation to *Mustelus* spp. [22,39]. This is the reason why fishers considered them to be not overfished and, in the case of *Squalus megalops*, fishers claim to “flee” from sites frequented by *S. megalops* as the longline catch ratio is 1:1. We know that bycatch cannot be entirely eliminated, so fishers have a role in handling and releasing sharks [11], even more so if significant injuries to the animals from hooks have occurred previously [11,40].

Although fishers have not observed species displacement due to anthropogenic causes, some claim to have noticed some species movement mainly due to water pollution, as well as abiotic factors such as temperature and depth as indicated by some studies [5,38], either in search of food such as small fish and invertebrates [41,42], to avoid predators [43], or because they prefer warmer waters [44]. In any case, they claim that individuals approach the coast in summer in accordance with mating or to give birth to their newborn specimens.

Sharks are characterised by K-type life strategies, that is, they grow slowly, reach sexual maturity late and have low fertility, making them more vulnerable to the consequences of overfishing, which can lead to population collapse in the long term. In order to conserve the species, future in-depth studies could propose seasonal closures to protect the reproductive season, more comprehensive regulation of minimum and maximum catch sizes, the establishment of marine protected areas, and uniformity in fishing statistics to be able to establish more clearly which species are being landed.

In conclusion, the results represent a relevant advancement, while acknowledging that future studies may expand and complement the evidence presented here. As well as infographics to train and raise awareness in the fishing sector about the taxonomic identification of species, not only for fishing and statistical purposes, but also to avoid as far as possible by-catches and reduce fishing mortality. Therefore, increasing species knowledge among the sector could bring us even closer to the current state of these fishery resources to achieve adequate conservation strategies.
